# The Combined Effects of Obesity and Cardiorespiratory Fitness Are Associated with Response Inhibition: An ERP Study

**DOI:** 10.3390/ijerph18073429

**Published:** 2021-03-25

**Authors:** Lin Chi, Chiao-Ling Hung, Chi-Yen Lin, Tai-Fen Song, Chien-Heng Chu, Yu-Kai Chang, Chenglin Zhou

**Affiliations:** 1 School of Physical Education, Minnan Normal University, Zhangzhou 363000, Fujian, China; chilin1215@hotmail.com; 2 Department of Athletics, National Taiwan University, Taipei 106319, Taiwan; musehung@gmail.com; 3 Physical Education Office, National Taiwan Ocean University, Keelung 202301, Taiwan; evalin@ntou.edu.tw; 4 Department of Sport Performance, National Taiwan University of Sport, Taichung 404401, Taiwan; tiffanyfen628@gmail.com; 5 Department of Physical Education, National Taiwan Normal University, Taipei 106209, Taiwan; 6 Institute for Research Excellence in Learning Science, National Taiwan Normal University, Taipei 106209, Taiwan; 7 School of Psychology, Shanghai University of Sport, Shanghai 200438, China

**Keywords:** body mass index, fitness, executive control, event-related potential

## Abstract

Obesity and cardiorespiratory fitness exhibit negative and positive impacts, respectively, on executive function. Nevertheless, the combined effects of these two factors on executive function remain unclear. This study investigated the combined effects of obesity and cardiorespiratory fitness on response inhibition of executive function from both behavioral and neuroelectric perspectives. Ninety-six young adults aged between 18 and 25 years were recruited and assigned into four groups: the high cardiorespiratory fitness with normal weight (NH), high cardiorespiratory fitness with obesity (OH), low cardiorespiratory fitness with normal weight (NL), and low cardiorespiratory fitness with obesity (OL) groups. The stop-signal task and its induced P3 component of event-related potentials was utilized to index response inhibition. The participants with higher cardiorespiratory fitness (i.e., the NH and OH groups) demonstrated better behavioral performance (i.e., shorter response times and higher accuracy levels), as well as shorter stop-signal response times and larger P3 amplitudes than their counterparts with low cardiorespiratory fitness (i.e., the NL and OL groups). The study provides first-hand evidence of the substantial effects of cardiorespiratory fitness on the response inhibition, including evidence that the detrimental effects of obesity might be overcome by high cardiorespiratory fitness.

## 1. Introduction

The obesity epidemic is increasingly regarded as a global pandemic, as more than 13% of people aged 18 years and over were of excessive weight in 2016 [1]. Obesity is not only linked to a broad range of long-term medical complications, such as cardiovascular disease, type II diabetes, and several types of cancers [2], but is also considered to be a risk factor for healthy lifestyle habits [3] and psychiatric conditions [4], such that it has profound economic consequences.

Obesity also has negative effects on various aspects of executive function (EF) [5], such as inhibition. Inhibition refers to the ability to override a planned, prepotent response or to stop already initiated responses (i.e., response inhibition) [6]. Several studies have utilized a highly theoretically driven cognitive task, the stop-signal task (SST) [7], to assess the efficiency of response inhibition. During the SST, the person being tested is required to response quickly to the go stimuli. Occupationally, the person needs to launch the stop-process induced by the stop stimuli to inhibit the go-process induced by the go stimuli, with that stoppage involving the top-down initiation of the aphasic inhibitory process [7]. Notably, the excessive weight has been attributed to failures to inhibit impulsive or prepotent responses [8]. For instance, individuals with higher BMI scores required longer duration of the reaction time to stop the ongoing response (i.e., stop-signal response time, or SSRT) compared to individuals with normal BMI scores [9]. Similarly, obese young adults have been reported to exhibit greater difficulties in response inhibition, as reflected by having longer SSRTs than normal-weight individuals [10], suggesting a relationship between less efficient response inhibition and obesity in the adult population. 

Cardiorespiratory fitness (CRF) may also affect various aspects of EF [11,12]. Cross-sectional research has revealed a positive relationship between CRF and the performance of the tasks involving interference aspect of inhibition in late-middle-aged [13] and older adults [14]. Similarly, studies using laboratory-based and filed-based CRF assessments have indicated positive associations between CRF and task performance involving response inhibition in preadolescent children [15] and young adults [16,17]. Finally, similar positive links between CRF and working memory [11,18] and shifting [18,19] aspects of EF have been reported.

The potential effect of CRF on the association between obesity and EF has been further suggested by the “fat-but-fit” paradigm. Specifically, the fat-but-fit paradigm suggests that higher levels of physical activity or CRF might alleviate some of the adverse effects of obesity (i.e., its effect on all-cause mortality) [20,21]. The fat-but-fit paradigm has been extended to attenuation of the adverse effects of obesity on cognitive function. For instance, Song, et al. [22] compared the performance of interference aspect of inhibition in relation to levels of CRF and BMI, and reported no significant differences in behavioral performance among young adults with high CRF, regardless of their weight status, on the neutral condition of the Stroop task, suggesting that CRF might alleviate the adverse effects of obesity on basic information processing. In another study, Ross, et al. [23] measured CRF, % of body fat, waist-height ratio, and cognitive function in adolescents, and found that the association between obesity and visual working memory was partially mediated by CRF. These studies have explored the interrelationships between CRF, obesity, and various aspects of EF (i.e., the interference aspect of inhibition and working memory), and have provided initial evidence supporting the conclusion that CRF might act to modulate, attenuate, or possibly offset the detrimental effects of obesity in some aspects of EF. Nevertheless, to the best of our knowledge, no research has investigated the interrelationship between CRF and obesity, in terms of their combined effects on response inhibition. 

Event-related potential (ERP), a noninvasive measure of brain electrical activity, may provide more insights regarding how response inhibition is associated with CRF and obesity. ERPs represent time-locked neuroelectric activities with sensitive temporal resolution, providing the opportunity for direct and detailed examinations of the neural mechanisms of mental processes [24]. In previous studies, individuals with higher CRF demonstrated larger P3 amplitudes along with shorter response times (RTs) and/or higher response accuracy levels [22,25,26]. Given that the P3 amplitude is regarded as a reflection of the amount of attentional resources allocated [27], such results suggest that higher CRF levels might support the recruitment of attentional resources and contribute to superior behavioral performance. Meanwhile, decreased P3 amplitudes and impaired response inhibition have been reported during the auditory discrimination task paradigm [28] and the Go/NoGo task [29] in obese and/or overweight children. 

Taken together, the above findings indicate that obesity and CRF are negatively and positively associated with inhibition, respectively. Nevertheless, the combined effects of CRF and excessive weight on response inhibition remain unknown. The present study was thus conducted to investigate how CRF and excessive weight are simultaneously associated with response inhibition, as assessed by the SST, from both behavioral and neuroelectric perspectives. Given the negative impacts of obesity and the positive effects of CRF reported previously, it was hypothesized that individuals with high CRF and normal weight would exhibit superior response inhibition-related performance and that obese individuals with low CRF would exhibit worse response inhibition-related performance, both in terms of behavioral and neuroelectric measures.

## 2. Materials and Methods

### 2.1. Participants

Healthy males aged 18 to 25 years who met the following criteria were recruited: (a) normal or corrected-to-normal vision; (b) no color blindness; (c) no history of neurological disorders, psychiatric disorders, or any brain injury; (d) no current substance abuse; (e) able to complete a CRF assessment based on the Physical Activity Readiness Questionnaire (PAR-Q); (f) body mass index (BMI) within the normal-weight (BMI = 18.5–24 kg/m^2^) or within the obese range (BMI > 27 kg/m^2^) based on the normative BMI data for Taiwanese adults published by the Ministry of Health and Welfare of Taiwan; (g) CRF level either above the 65th percentile or below the 35th percentile, as reflected by the maximal oxygen uptake (VO_2max_) index, based on the norms provided by the American College of Sports Medicine [30]; and (h) right-handed. The experimental protocol for the study was approved by the Institutional Review Board of National Taiwan University. Following an initial screening, 92 eligible participants were grouped into four mutually exclusive groups: the high CRF with normal weight (NH, *n* = 23) group, high CRF with obesity (OH, *n* = 23) group, low CRF with normal weight (NL, *n* = 23) group, and low CRF with obesity (OL, *n* = 23) group. The demographic data and working memory aspect of the intelligence quotient of each participant, as assessed by the Forward and Backward Digit Span Test of the Wechsler Adult Intelligence Scale-Third Edition [31], were collected.

### 2.2. Submaximal Cardiorespiratory Fitness Assessment

All of the participants completed the YMCA cycling test [32], which is a submaximal CRF test and has been described as effective in predicting VO_2max_ [33]. The test consists of three consecutive 3-min cycling stages. For each participant, the test began with a 3-min warm-up stage involving exercising on a braked cycle ergometer (Ergoselect 100/200 Ergoline GmbH, Germany) at 150 kpm/min (25 W) and pedaling at a constant speed of 50 rpm. The subsequent stages of power output progression were determined by the given participant’s steady-state HR recorded during the last 15–30 s of the initial 3-min warm-up stage. An additional 3-min stage was added if the participant’s target HR (i.e., 85% of the individual’s age-predicted HRmax) was not achieved. The result for the Borg Rating of Perceived Exertion (RPE) scale [34] was recorded at the end of every 3-min stage during the exercise period. Finally, each participant’s VO_2max_ was predicted using the extrapolation method.

### 2.3. Stop-Signal Task (SST)

The SST conducted using the e-prime software was adopted from Johnstone, et al. [35]. The go-trials of the SST consist of leftward- or rightward-pointing black arrows (← or →) with 196 × 42 pixels (hereafter referred to as go stimuli). The probability of a leftward- or rightward-pointing arrow occurring for any trial was 50%. A plus sign was presented as the fixation for 500 ms at the beginning of each trial. Following the plus sign, a blank screen (100 ms) and the go stimuli (500 ms) were presented sequentially. Participants were instructed to press the response button on the response box corresponding to the direction of each presented arrow (that is, they were asked to press the left response button for a leftward-pointing arrow and the right response button for a rightward-pointing arrow) as quickly and accurately as possible.

When presented, the stop stimulus (i.e., a red square superimposed on the go stimulus) was displayed with a certain delay (i.e., the stop-signal delay, SSD) following the go stimulus onset. The given participant was asked to withdraw their response when they detected the presence of the stop-signal stimulus (with each trial involving the stop-signal stimulus hereafter referred to as a stop-trial). The SSD was initially set to 200 ms, and dynamically varied between 50 ms and 450 ms according to each participant’s ongoing performance. Specifically, the SSD was increased by 50 ms if the participant could successfully withdraw their response during the previous stop-trial and decreased by 50 ms if the participant failed to withdraw their response during the previous stop-trial. With the dynamic tracking algorithm, success in withholding the response was achieved for approximately 50% of the stop-trials. Presentation of the go- and stop-trials was randomized to prevent subjective expectancy. A low proportion of stop signals (i.e., 25%) was chosen for the trials in order to increase the strength of the conflicting stimuli. Finally, the SSRT of each participant was calculated by subtracting the participant’s average SSD from their average response time during the go-trial (i.e., the mean go-trial RT).

### 2.4. Psychophysiological Recording and Data Analysis

For each participant, the brain electrical activity was recorded continuously from 32 Ag/AgCl scalp electrodes of the international 10-20 system inserted in an elastic cap (Quick-Cap, NeuroScan Inc.) and referenced to the left and right mastoids. The horizontal electrooculogram (HEOG) was recorded from two electrodes attached laterally to each eye to monitor horizontal eye movements. Vertical eye movements and blinks (the VEOG) were recorded from two electrodes attached above and below the left eye. The electrode located on the mid-forehead was used as the ground electrode. The impedance of all the electrodes throughout the recording period was maintained below 10 kΩ. The online EEG signal was digitized at a rate of 500 Hz and amplified by a SynAmps EEG amplifier filtered with a bandpass between 70 Hz and 0.05 Hz and with a 60-Hz notch filter to remove additional electrical noise with the NeuroScan equipment (NeuroScan Inc., El Paso, TX, USA).

The offline EEG data was then analyzed using the Scan software (NeuroScan Inc., El Paso, TX, USA, v4.5). Only the successfully inhibited and corrected response trials were included for further analysis. The offline EEG data was initially processed by correcting the eye movements and blinks using the algorithm proposed by Semlitsch, et al. [36], and an epoch of 1300 ms was segmented starting 100 ms prior to the onset of the go stimulus and lasting until 1200 ms after the go stimulus onset for each trial. The average artifact-free epoch waveforms were computed for both go-trials and stop-trials. The data was then digitized and filtered with a bandpass filter between 0.05 and 30 Hz (12 dB/Oct). The EEG data from poorly recorded channels and trials for which the amplitude exceeded ± 85 μV was excluded from further analysis.

ERP averages were then computed for the successful stop-trials and the correct go-trials for each individual. The initial time windows for the go-P3 (200–700 ms) component during the go-trials were determined based on the grand average waveforms of the four groups. The time windows for the stop-ERP components were determined from the average stop-signal onset time with the consideration of variation in each individual’s SSD. That is, the final lower and upper time windows for each participant were equal to the smallest SSD plus the lower limit of the average stop-signal onset time and the largest SSD plus the upper limit of the average stop-signal onset time, respectively. Finally, for each individual P3 time window, the P3 amplitude, defined as the most positive value within the window, was identified by means of an automatic pick-picking program using the Scan software (NeuroScan, v4.5). In a similar manner, the N2 amplitude, which was defined as the most negative value within the final time window, was also identified.

### 2.5. Experimental Procedure

The participants were required to visit the laboratory located on the National Taiwan Sport University campus individually on two occasions separated by less than seven days from each other. In order to measure the body-weigh accurately, all participants were instructed to avoid having any food for 8 h prior their body-weight measurements. Additionally, they were informed not to consume any food or drink containing caffeine or alcohol and to avoid engaging in any strenuous exercise for 12 h prior to second experimental session in an effort to reduce any effect of stimuli on the cardiovascular system. 

During the first visit, the experimental procedure was introduced by the researchers conducting the experiment, and informed written consent was obtained from the given participant. After the informed consent was completed, information regarding the participant’s health/physical condition was acquired by filling out the demographic and PAR-Q questionnaires. The participant’s BMI was then calculated based on their weight (kg) and height (m), and the participant’s score on the Digit Span Test was recorded. Following confirmation that the health condition and BMI status of the participant met the inclusion criteria, the participant was fitted with a Polar heart rate monitor (Sport Tester PE 3000, Polar Electro Oy, Kempele, Finland) and instructed to perform the YMCA cycling test at a room temperature of 20 °C. The resting HR (HR rest) of the participant was measured after the participant sat still for 5 min. The participant’s HR during the exercise was recorded every 2 min. Those participants who met the inclusion criteria were invited to return for the second experimental session.

During their second visit, each qualified participant was seated comfortably in a chair in a dimly lit, sound-attenuated, air-conditioned, and electrically shielded room where they then completed the SST. The viewing distance between the participant and the computer screen was set at 70 cm. During the SST, the participant was instructed to focus on a central fixation cross on the screen and to avoid making any body movements during the EEG recording. Prior to being tested with the experimental blocks of the SST, the participant responded to a practice block of 10 trials that allowed the participant to become familiar with the task so that they could keep in mind that they should inhibit their response when the stop signals appeared in the stop-trials. Furthermore, the importance of responding quickly to the go stimuli was emphasized, and the participant was told not to sacrifice response speed by waiting for the occurrence of the stop signals. Time was taken to ensure that each participant clearly understood the test prior to the experimental blocks being presented. After the practice block, two experimental blocks, each containing 200 trials, were presented. A 5-min break was taken halfway through each block, and a 15-min break was taken between the two blocks. EEG recordings were taken throughout the task performance period. The duration of the experiment was about 1.5 h. Each participant received a payment of approximately US $30 for travel expenses incurred.

### 2.6. Statistical Analysis

The demographic data was analyzed using the one-way analysis of variance (ANOVA) among the four groups (i.e., the NH, OH, NL, and OL groups). For behavioral data, the ANOVA was also conducted for the go RTs, which were defined as the time intervals between the onsets of the go stimuli and the correct responses made by the participants; the accuracy of the go-trials; and the SSRTs. For electrophysiological data, a separate ANOVA was also conducted for the go- and stop-P3 amplitudes at the parietal region (the averaged ERP from the P3, Pz, and P4 electrode locations). Finally, post-hoc Tukey HSD and multiple t-test comparisons with Bonferroni correction were conducted where appropriate. All statistical analyses were carried out using SPSS (version 21.0, IBM, Corp., Armonk, NY, USA). Mean and standard error (SE) values were presented.

## 3. Results

### 3.1. Participant Characteristics

The demographic background data of the participants is summarized in Table 1. The ANOVA revealed that there were no significant differences among the four groups in terms of age (years), height (cm), or working memory performance.

In terms of weight status (kg), significant differences between the groups were observed (*p*s < 0.05). Follow-up analyses indicated that the OL group was significantly heavier than all three other groups (*p*s < 0.05), while the OH group was significantly heavier than the NH and NL groups (*p*s < 0.01). Meanwhile, no difference in weight status between the NL and NH groups was observed. In terms of BMI scores, significant differences between the groups were observed (*p*s < 0.05). Follow-up analyses indicated that the OL group had significantly higher BMI scores than the other three groups (*p*s < 0.01), while the OH group had higher BMI scores than the NL and NH groups (*p*s < 0.01). No differences were observed, meanwhile, between NL and NH groups.

With regard to the CRF levels, significant differences in VO_2max_ scores were observed among the groups (*p*s < 0.05). Follow-up analysis indicated that both the NH group and the OH group had higher VO_2max_ scores than the NL group, and that the OL group had the smallest VO_2max_ scores.

### 3.2. Behavioral Data

**Figure 1:**
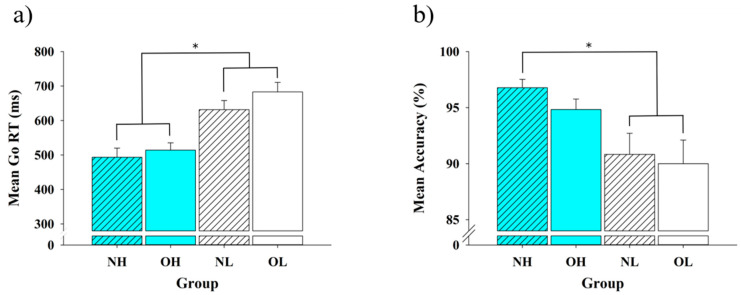
A comparison of the (**a**) response time (RT) and the (**b**) accuracy (means ± SE) on the Stop-Signal task during go-trials for the four groups. NH = High CRF with normal weight; OH = high CRF with obesity; NL = low CRF with normal weight; and OL = low CRF with obesity. * *p* < 0.05.

Response time: One-way ANOVA analysis revealed a main effect of group [*F* (3, 88) = 12.78, *p* < 0.01] (Figure 1a). The follow-up analysis revealed that both the NH group (493.54 ± 26.39 ms) and the OH group (514.27 ± 20.99 ms) had significantly shorter RTs than the NL and OL groups (683.00 ± 27.87 ms and 631.54 ± 26.58 ms, *p*s < 0.05). No significant differences were observed, however, between the NH group and the OH group, or between the NL group and the OL group.

Accuracy: One-way ANOVA analysis revealed a main effect of group [*F* (3, 88) = 4.41, *p* < 0.01] (Figure 1b). The follow-up analysis revealed that the NH group (97 ± 0.75 %) had a significantly higher accuracy rate than the NL and OL groups (90 ± 2.1 % and 91 ± 1.9, *p*s < 0.05). No other significant differences among the OH, the NL, and the OL groups were observed (*p* > 0.05).

**Figure 2:**
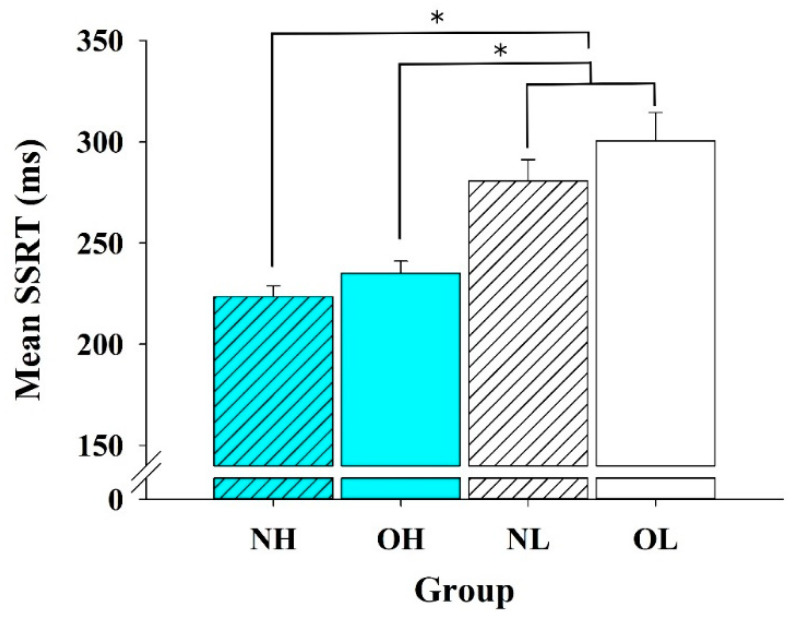
Comparisons of the stop-signal response time (SSRT) across the four groups (means ± SE). NH = High CRF with normal weight; OH = high CRF with obesity; NL = low CRF with normal weight; and OL = low CRF with obesity. * *p* < 0.05.

SSRT: One-way ANOVA analysis revealed a main effect of group [*F* (3, 88) =14.648, *p* < 0.01] (Figure 2). The post-hoc analysis revealed that both the NH group (223.55 ± 5.31 ms) and the OH group (235.15 ± 5.98 ms) exhibited shorter SSRTs than the NL and OL groups (300.55 ± 13.91 ms and 280.82 ± 10.42 ms, *p*s < 0.05). Furthermore, the mean SSRT of the OH group (235.15 ± 5.98 ms) was also significantly shorter than the mean SSRTs of the OL and NL groups (*p* < 0.05).

### 3.3. ERP Data: P3 Amplitudes

**Figure 3:**
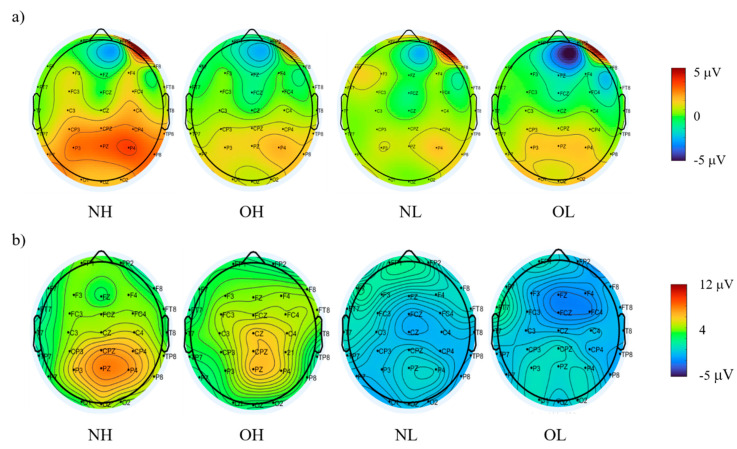
The topographic distributions of the P3 amplitude of (**a**) go- and (**b**) stop-trials across four groups. NH = High CRF with normal weight; OH = high CRF with obesity; NL = low CRF with normal weight; and OL = low CRF with obesity.

Regarding the go-P3 amplitude, one-way ANOVA analysis revealed a main effect of group [*F* (3, 88) = 4.02, *p* < 0.05], with the NH group (7.05 ± 0.94 μV) having a significantly larger mean amplitude than the OH (4.34 ± 0.64 μV), NL (4.34 ± 0.64 μV) and OL (3.99 ± 0.56 μV) groups. There was no significant difference among the OH, NL, and OL groups (*p* > 0.05). Regarding the stop-P3 amplitude, a main effect of group was also revealed [F (3, 88) = 5.71, *p* < 0.01], with the NH group (16.68 ± 1.26 μV) having a significantly larger mean amplitude than those observed for the NL (11.18 ± 0.79 μV) and OL (10.13 ± 1.28 μV) groups. No significant difference between the NH group and the OH group (12.55 ± 1.40 μV) group was observed. The topographic distributions of the P3 amplitude of go- and stop-trials across four groups was also presented in Figure 3.

## 4. Discussion

The purpose of the current study was to examine the interrelationship between CRF and excessive weight in terms of their combined effects on response inhibition induced by the SST from both behavioral and ERP perspectives. Our primary behavioral findings revealed that the participants with high CRF levels, regardless of their normal or excessive weight status, had superior response inhibition in comparison to the participants with low CRF levels. Furthermore, the individuals with normal weight and high CRF demonstrated a higher accuracy rate than the other three groups. With regard to ERP indices, the individuals with normal weight and high CRF exhibited significantly larger go- and stop-P3 amplitudes than the obese individuals with low CRF levels, while no significant differences in the P3 amplitudes were observed among the other three groups.

### 4.1. Behavioral Performance

Superior performance under time pressure reflected by faster response times (in terms of go RTs) was observed among the participants with high CRF (i.e., the NH and OH groups) in comparison to the participants with low CRF (i.e., the NL and OL groups). These findings are consistent with those of prior research reporting that young adults with higher fitness levels had shorter go RTs during the neutral condition of the Stroop task [22], and the congruent condition of the Erickson flanker task [37]. However, similar go RTs for active and non-active young adults have also previously been observed [16,17], which is somewhat inconsistent with our findings in the present study. This inconsistency may be linked to the categories of CRF levels used in the different studies. Specifically, the average CRF levels for the non-active participants in the study by Padilla and colleagues were 44.43 and 48.00 mL/kg/min, respectively, which were higher than those of the low-fitness participants in the current study (35.79 and 36.45 mL/kg/min for the NL and OL groups, respectively). That is, our findings might represent the influence of relatively low CRF levels on the go RT. Given that go RT reflects the time required for the generalized processing needed for stimulus identification, stimulus discrimination, response choice, and response execution [38], CRF may have a positive influence on the general processing speed relative to the weight status during the SST. 

Our SSRT results revealed that one difference between the high- and low-fitness participants was in response inhibition ability, with the shorter SSRTs of the participants with high CRF indicating their superior response inhibition compared to their low CRF counterparts. Such superior inhibition on the part of the high CRF participants was in line with previous studies regarding CRF. For example, previous studies have reported that active individuals demonstrated shorter SSRTs in the more executive control-demanding version of the SST compared to their inactive counterparts [16,17]. Other studies using the Erickson flanker task also found that higher CRF is associated with outcomes indicating superior inhibition, such as smaller flanker effects in older adults [39,40], better ability to maintain response accuracy across task conditions [25], and less response variability [41] in children. Consistent with this positive association, prior research has further reported superior performance in the incongruent condition of the Stroop task among older [42] and young adult [22] populations with higher fitness levels. 

The covert behavioral response of SST (i.e., success or failure to withhold the on-going go-process) could be interpreted by the “horse-race” model [7], which indicates that the stop performance is determined by the “race” between the go-process and stop-process; that is, a participant will not be able to withhold or terminate their pre-potent response if the “go” process finishes the race before the "stop" process does (i.e., when go RT < (SSRT + SSD)) [7]. Accordingly, successful withholding of the ongoing go-process could be achieved by decreasing the tendency toward executing covert behavioral responses, through either or both of slowing the go RT and shortening the SSRT, for a given SSD [43]. Notably, although faster general cognitive processing speed (i.e., shorter go RTs) observed in individuals with higher CRF (i.e., NH and OH group) might imply a higher tendency toward executing the ongoing go-process, the superior response inhibition (i.e., shorter SSRTs) in the NH and OH groups might suggest greater effects of CRF on the processing of stop-signal detection and the initiating of the response inhibition. Additionally, the higher accuracy rates and shorter go RTs demonstrated by the NH group further indicate that this superior performance was not caused by a speed-accuracy trade-off during the task performance. Taken together, and in addition to the prior findings indicating a positive association between CRF and inhibition, the data collected in the current study thus provide further evidence indicating a beneficial effect of CRF on response inhibition.

Contrary to our predictions, however, our results revealed no differences in response inhibition across weight categories in terms of SSRT performance. While negative associations between the standard SSRT and obesity in adults [10,44] and children [45,46,47] have previously been reported, non-significant differences in SSRT between normal weight and obese individuals have also been observed [44,48,49,50]. These inconsistent findings might result from the types of stimuli used [51], as well as the association between obesity and EF being altered by CRF. For instance, Edwards, Dankel, Loenneke and Loprinzi [21] reported that lower executive performance was only related to the present inactivity of older adults, regardless of individuals’ past and current weight statuses. Our previous findings also suggested that the inhibition levels assessed by the Stroop task were similar for high-fitness participants with normal weight and high-fitness participants with obesity [22]. As such, the results of the present study extend the current understanding of the interrelationship between CRF and obesity in terms of their combined effects on EF, as well as further supporting the fat-but-fit paradigm in relation to response inhibition.

### 4.2. ERP

Larger stop-P3 and go-P3 amplitudes were observed in the participants in this study with normal weight and high fitness (i.e., the NH group) than in those with normal weight and low fitness and those with obesity and low fitness (i.e., the NL and OL groups). This observation of a fitness-related difference in P3 amplitudes corroborates the current literature regarding fitness and response inhibition in young adult populations. Specifically, larger P3 amplitudes were previously observed for normal-weight young adults with high CRF levels than for their low-fitness counterparts, along with superior performance across both the neutral and incongruent conditions of the Stroop task [22]. A similar pattern has been reported in preadolescent children, that is, larger P3 amplitudes and superior behavioral performance have been seen in high-fitness as compared to low-fitness healthy preadolescent children [25,26]. 

Stop-P3 amplitude has previously been reported to be related to the active inhibitory processing of motor responses released by the stop signal [52,53]. Based on the reduced P3 amplitudes in children with attention deficit/hyperactivity disorder during the Erickson flanker task [54] and during the SST [55], it has been suggested that stop-P3 amplitudes involve monitoring of the outcomes of the inhibitory process and its efficiency. In consequence, the larger stop-P3 amplitudes among individuals in the NH group in the current study might reflect a surplus in cognitive control abilities affecting overall performance monitoring. This hypothesis is supported by a previously reported positive association between CRF and the volume of the anterior cingulate cortex (ACC) and reduced ACC activity reflecting better conflict monitoring after aerobic exercise interventions [40,56], as well as studies linking ACC activity with the monitoring of go and stop performance in response inhibition [43]. Therefore, the enhanced modulation of the P3 component observed in the NH group in this study may result from functional and structural changes of the ACC. 

P3 amplitude has also been associated with the amount of attentional resources allocated during a cognitive task [27] as well as enhanced go response processing [53]. As a result, the larger stop- and go-P3 amplitudes observed in the NH group might reflect a superior ability to allocate larger amounts of attentional resources, less impulsivity, and faster processing speed for go signals. Given the shorter go RTs and SSRTs, as well as the findings regarding P3 amplitudes, observed for the NH group, it is conceivable that the higher fitness levels of this group cooperated with their normal weight statuses to provide superior abilities in terms of several neuroelectrical processes related to both overall processing speed as well as inhibition.

It is important to note that, while the individuals in the NH group demonstrated the largest P3 amplitudes compared to the participants in the low-fitness groups, the P3 measure itself partly consisted of behavioral performance; that is, despite the OH group demonstrating similar overt stopping performance compared to the NH group, the mean P3 amplitude of the OH group was attenuated and did not differ significantly from those of the NL and OL groups. This finding might indicate that the OH group had, in comparison to the NH group, less attentional resource allocation and/or weaker activation levels that were not observable through their overt behavior alone. Additionally, the finding of no difference in P3 amplitudes for the NL group compared with OH and OL groups was partially consistent with of our prior study [22], in which no difference in behavioral results and P3 amplitudes between NL, OH, and OL groups was reported. However, the findings from both studies might suggest that young adults with normal weight and low CRF levels have lower levels of response inhibition, at least from the neuroelectrical perspective, similar to those of the obese population.

There are several potential mechanisms through which higher CRF might alleviate the adverse effects associated with obesity. Firstly, the reduction of cerebral blood flow associated with obesity [57] might be countered by the improved cerebral blood flow regulation resulting from exercise and/or higher CRF, as both exercise and higher CRF have been associated with better cerebral blood flow regulation in young adults [58] and older adults [59]. Another mechanism might be related to the ameliorating effects of CRF on the chronic systematic inflammation associated with obesity [60]. This systematic inflammation might lead to neuroinflammation in certain brain regions (i.e., the hypothalamus), and inflammatory biomarkers (i.e., interleukin-1 beta) have also been associated with the suppression of brain-derived neurotrophic factor signaling [61], which has vital importance in neuronal repair, neuronal survival, neurogenesis, and neuroplasticity [62]. In contrast, CRF has been associated with reduced levels of inflammatory biomarkers and enhanced BDNF levels [63]. Therefore, CRF might alleviate the adverse effects of obesity and ultimately enhance cognitive function. Finally, prior research has also provided evidence of an association between higher CRF with larger brain volumes (i.e., prefrontal cortex, basal ganglia, and caudate nucleus volumes) and superior cognitive function [64,65,66]. This might also contribute to alleviation of the negative impacts of obesity on brain volumes and cognitive function [67,68,69].

### 4.3. Limitations and Future Directions

Due to various limitations, the findings of the current study should be interpreted with some caution. Firstly, the study’s cross-sectional design prevents any interpretations regarding causality [70]. However, the current study took an initial step in identifying the interrelationships among CRF, obesity, and response inhibition, thereby providing a basis from which to determine the associated cause-effect relationships. Another limitation of this study, meanwhile, is related to its generalizability in terms of population given that only young male adults were recruited. Females have been demonstrated to have superior behavioral inhibitory performance, as well as shorter latencies and larger amplitudes in several ERP components, than males [71], implying that gender differences should also be taken into consideration. Finally, the establishment of obesity status in this study was based only on BMI scores, and although BMI scores have been widely utilized in studies of obesity, BMI is a measure of body fatness rather than exact body fat. As such, measures of body composition or biochemical indices might provide more accurate classifications of obesity and reveal different results [72].

## 5. Conclusions

The current study provides empirical evidence regarding the interrelationships among CRF, obesity, and response inhibition. Additionally, by employing ERPs, it allowed us to further identify the potential underlying neuronal mechanisms. In brief, young adults with higher levels of CRF, regardless of their weight status, were found to exhibit superior performance in terms of response inhibition. Moreover, the high CRF participants were also observed to have superior attentional resource allocation and inhibitory processing speeds, as well as higher efficiency in terms of the executive control system. Future research employing randomized controlled trials is thus warranted to examine how CRF and weight status changes might be related to the degree of EF benefits achieved.

## Figures and Tables

**Table 1:** Demographic and cardiorespiratory fitness for participants (means ± SD).

Variables	NH (*n* = 23)	OH (*n* = 23)	NL (*n* = 23)	OL (*n* = 23)
Age (years)	20.52 ± 1.65	20.70 ± 2.16	21.47 ± 2.00	21.04 ± 2.16
Height (cm)	173.43± 4.86	177.26 ± 7.63	175.91 ± 4.74	174.30 ± 6.27
Weight (kg)	63.91 ± 5.28	91.78 ± 14.54 ^b^	66.65 ± 5.36	102.43 ± 19.93 ^a^
BMI (kg/m^2^)	21.25 ±1.38	29.08 ± 2.50 ^b^	21.53 ±1.23	33.63 ± 5.93 ^a^
Digit span: Forward	14.50 ± 1.30	14.00 ± 1.43	14.68 ± 1.32	14.14 ± 1.08
Digit span: Backward	8.82 ± 3.10	8.23 ± 2.10	9.60 ± 2.67	8.55 ± 2.76
VO_2max_ (mL/kg/min)	55.19 ± 4.73 ^a^	53.28 ± 4.29 ^a^	41.66 ± 9.60	36.04 ± 3.63

NH = High cardiorespiratory fitness (CRF) with normal weight; OH = high CRF with obesity; NL = low CRF with normal weight; and OL = low CRF with obesity. ^a^ and ^b^ = *p* < 0.05.

## Data Availability

All datasets generated for this research are included in this published article.
